# Large scale comparison of global gene expression patterns in human and mouse

**DOI:** 10.1186/gb-2010-11-12-r124

**Published:** 2010-12-23

**Authors:** Xiangqun Zheng-Bradley, Johan Rung, Helen Parkinson, Alvis Brazma

**Affiliations:** 1 European Bioinformatics Institute, Wellcome Trust Genome Campus, Cambridge, CB10 1SD, UK

## Abstract

**Background:**

It is widely accepted that orthologous genes between species are conserved at the sequence level and perform similar functions in different organisms. However, the level of conservation of gene expression patterns of the orthologous genes in different species has been unclear. To address the issue, we compared gene expression of orthologous genes based on 2,557 human and 1,267 mouse samples with high quality gene expression data, selected from experiments stored in the public microarray repository ArrayExpress.

**Results:**

In a principal component analysis (PCA) of combined data from human and mouse samples merged on orthologous probesets, samples largely form distinctive clusters based on their tissue sources when projected onto the top principal components. The most prominent groups are the nervous system, muscle/heart tissues, liver and cell lines. Despite the great differences in sample characteristics and experiment conditions, the overall patterns of these prominent clusters are strikingly similar for human and mouse. We further analyzed data for each tissue separately and found that the most variable genes in each tissue are highly enriched with human-mouse tissue-specific orthologs and the least variable genes in each tissue are enriched with human-mouse housekeeping orthologs.

**Conclusions:**

The results indicate that the global patterns of tissue-specific expression of orthologous genes are conserved in human and mouse. The expression of groups of orthologous genes co-varies in the two species, both for the most variable genes and the most ubiquitously expressed genes.

## Background

Over the past two decades, both tissue specificity and the conservation of expression between orthologous genes have been much discussed but comparative analysis at the transcriptome level has produced ambiguous results. While studies suggested that orthologous genes do not share similar expression patterns [1-5], other groups reported the opposite observations [6-9]. In fact, gene-specific expression regulation is different in mouse and human. For instance, it has been shown that even for highly conserved and tissue-specific transcription factors, promoter-binding events are highly species specific, and binding patterns do not align between species [10]. We took advantage of the vast amount of human and mouse gene expression data deposited in ArrayExpress to investigate possible correlation of global patterns between mouse and human orthologous genes at the expression level.

The challenge of comparing expression patterns of orthologous genes in different species is mainly due to different affinities of probes on different chips, leading to difficulties in comparing data from different platforms. Different approaches have been tried to compare gene expression patterns in different organisms (reviewed in [11]). Some studies used the same microarray for cross-hybridization in samples from different species to eliminate the variations in hybridization and scanning protocols. This approach typically used either a single-species array, to which samples from closely related species or subspecies were hybridized and expression levels of orthologous genes were measured [12,13], or a custom-designed chip that contained probes from different species [14,15]. Alternatively, many other studies made use of species-specific arrays to identify co-expressed groups of orthologous genes [4-6,16,17]. In such studies, how to minimize the platform effects was the key to meaningful comparison of the cross-species data. Some studies identified differentially expressed genes within species; then the resulting significant gene lists were compared cross-species to look for patterns of conservation [3,18]. A few other studies used more sophisticated algorithms and analyzed combined data from different species at the same time to identify cell cycle genes with conserved expression patterns between species [19-21].

Our study used data generated on species-specific microarray platforms. Only human data from the Affymetrix HG-U133A array and mouse data from the Affymetrix MG_U74Av2 array were considered to exclude between-array variability within each species. These two whole genome arrays were selected because they have been used for the highest number of human and mouse samples in ArrayExpress. Raw data consisting of 5,372 and 1,323 high quality human and mouse CEL files were selected from ArrayExpress. Each CEL file corresponds to the hybridization of one biological sample. Since the data matrices are extremely large and the information content is very rich, we first normalized and filtered for human-mouse orthologous probesets, then used principal component analysis (PCA) to reduce the data dimensions. PCA has been often used to study high-dimensional data generated by genome-wide gene expression studies [22-25]. In an earlier PCA analysis of the 5,372 human hybridizations it was found that, on PCA scatter plots, samples in general clustered together based on tissue types. Despite the great diversity, the samples are predominantly clustered into the following classes of distinctive biological characteristics: hematopoietic system, malignancy samples including cell lines, neoplastic sample and non-neoplastic primary tissues, and nervous system. Specific classes of genes are expressed in different clusters [25]. The study suggested that samples of similar physiological attributes have similar gene expression profiles globally and they would tend to group together on PCA scatter plots.

It is intriguing whether these major gene expression patterns are conserved across evolutionarily diverse species such as human and mouse. We answer this question positively and report a similar PCA analysis of the 1,323 mouse hybridizations. Similar to what was observed in the previous study of human data [25], the mouse samples also clustered on PCA scatter plots. The samples were loosely partitioned into a nervous system cluster, a muscle/heart cluster, a liver cluster and a cluster of samples with lower variability, including cell line samples. Since the distribution of samples on the scatter plots is driven by the underlying transcriptome, we anticipate that samples in each cluster have distinctive gene expression profiles. To compare gene expression profiles between human and mouse, the data from the two species were normalized and merged into a single data matrix based on orthologous gene pairings. The merged data matrix was subjected to PCA analysis. We observed that the clustering of samples in individual species is well preserved in the multi-species analysis; more interestingly, human and mouse share a very similar pattern of sample clustering. The resemblance of the human and mouse sample clusters was also observed in hierarchical clustering of Pearson correlation between human and mouse tissues. All observations suggest that, for at least a fraction of orthologous genes, the expression profiles are largely conserved between the two species. The speculation is supported by elevated gene expression correlation co-efficient between human and mouse orthologous genes comparing with a randomized negative control. Additional investigations allowed us to identify orthologous genes whose expression levels co-vary in the two species.

## Results and discussion

### Sample clustering analysis of the mouse dataset

**Figure 1:**
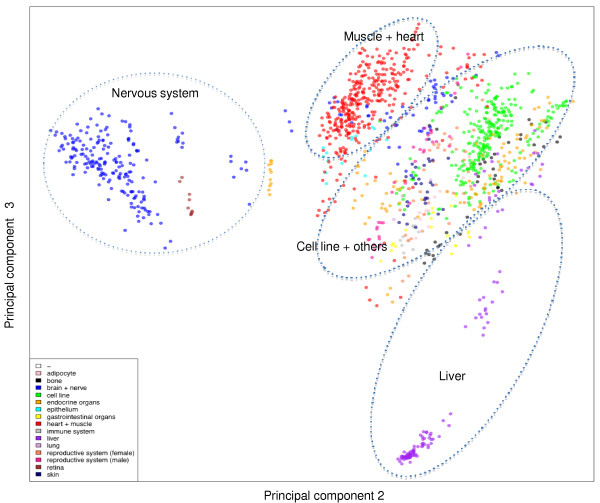
**PCA plot of the integrated mouse gene expression data matrix**. Each dot represents a sample, which is colored by the annotation of its tissue type. The samples can be loosely divided in four areas from left to right: nervous system (blue), muscle/heart (red), cell line (green) and others, and liver (purple). The brown dots co-clustering with nervous system samples are retina samples. Samples with unknown organism part (-) are white so they are invisible.

An integrated mouse gene expression dataset based on Affymetrix platform MG_U74Av2 was created as described in Materials and methods. It can be downloaded from the ArrayExpress website [26], accession number E-MTAB-27. The data matrix of E-MTAB-27 contains normalized gene expression measurements for 1,323 samples from 71 independent experiments for 12,488 probesets, which map to 8,741 genes with Ensembl identifiers (Table 1). To explore whether the 1,323 samples form distinct groups based on their gene expression profiles, the data matrix was subjected to PCA and the results are visualized by scatter plots. As shown in Figure 1, the majority of brain and nerve samples form a distinct group together with a number of retina samples. The retina and the optic nerve originate as outgrowths of the developing brain and are considered as part of the central nervous system, which can explain this co-clustering. Liver samples form a loose cluster compared to the denser nervous system cluster. The third dominant cluster consists of heart and muscle samples, and this co-clustering is not surprising considering that heart is composed mainly of cardiac muscles. A central cluster, denser than the three main tissue specific clusters, consists of cell lines and other less numerous samples, such as bone and immune system. This co-clustering of many sample types in the central PCA cluster, in particular the cell line samples, was observed in human studies [25] and may be due to a relatively small degree of correlation variability between samples. Cell lines of various tissue types are more homogeneous in their expression profiles than the original tissues, either because of less possible variability in the sample preparation, or because the immortalization procedure has had a profound effect on expression regulation.

**Table 1:** Summary of probesets and probeset annotations for the platforms used in the study

	Mouse	Human	Cross-species
Number of probesets	12,488	22,283	6,180
Number of annotated probesets	9,396	18,387	6,180
Number of Ensembl genes	8,741	13,199	5,925

Three platforms are listed: mouse platform MG_U74Av2, human platform HG-U133A and the reduced cross-species platform containing only orthologous probesets between human and mouse. Annotated probesets are those with gene annotations. The last row in the table is numbers of Ensembl genes represented by the probesets in each platform.

Further analysis demonstrated that samples of a particular tissue type are always represented by multiple experiments (Additional files 1 and 2), suggesting that lab effects did not drive the tissue clustering. We conclude that, similarly to what has been observed in human, mouse samples from a given tissue class share similar global gene expression patterns, causing the samples to cluster together when they are projected to the top principal components. When profiling the transcriptome of thousands of samples from different tissues and different conditions, the subtle variations within the same class of samples give way to the grand differences between different sample classes.

### Sample clustering analysis of combined human and mouse datasets

**Figure 2:**
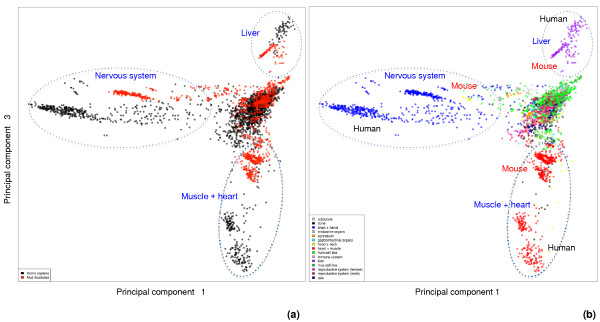
**PCA plots of a combined human and mouse gene expression data matrix (principal components 1 and 3)**. Each dot represents a sample, which is labeled by **(a) **species and **(b) **tissue type. Cell line samples from both species form a big central cluster, together with a relative small number of samples from immune system, reproductive system, bone, endocrine organs and other tissue sources from both species. Away from this central cluster, three major sample clusters are indicated: muscle/heart samples (red), nervous system samples (blue) and liver samples (purple). For these three clusters, human and mouse samples exhibit subclustering in proximity to each other. In the nervous system cluster, a few mouse head and neck samples (yellow) are mixed in - these are retina samples that have been generalized into the head and neck category. In the muscle/heart cluster, a few human bone samples (black) and a few head and neck samples (yellow) are mixed in.

To compare the expression pattern of human and mouse, a direct way is to put normalized expression data of the two species together and reduce the data complexity by PCA. On scatter plots of two principal components, will samples cluster by species or by tissue types? To answer this question, we created an integrated mouse and human gene expression matrix, containing 6,180 orthologous probesets measured for 3,824 samples (2,557 human and 1,267 mouse), as described in Materials and methods. The data can be downloaded from our web site [27] in the form of Bioconductor's ExpressionSet objects; a README in the same directory gives instructions on how to extract matrix of expression values and sample annotation from the R objects. The 6,180 probesets represent 5,925 Ensembl genes (Table 1). The samples for this analysis were selected to maintain a balance in tissue representation between mouse and human, to allow as much comparability between sample groups as possible between the two species. Samples prevailingly dominant in one species were removed from both species, which include all mammary gland and all blood and bone marrow samples. This process removed 2,815 human samples and 56 mouse samples from the raw datasets. The normalized human and mouse matrices were merged based on orthologous probesets; the merged matrix was then analyzed by PCA. When the data were normalized by probeset, the first three principal components explain more than half of the data variance (Additional file 3a). Scatter plots of components 1 and 3 are shown in Figure 2a,b, in which samples are labeled by species and tissue type, respectively.

In the combined analysis, we observe the same cluster pattern as in the mouse-only analysis. The four predominant groups are a central cluster of mostly cell line samples, and three tissue-specific clusters: muscle/heart, nervous system, and liver samples (Figure 2). Human samples and mouse samples form the same major clusters, and the tissue-specific clusters of samples from each species are adjacent in the PCA plot. Similar sample clustering patterns were observed in scatter plots of other principal components; one example is components 1 and 2 in Additional file 4. Since the distance between two samples when projected onto the principal components is determined by the covariance of their gene expression profiles, we believe the similarity of the human and mouse tissue clusters reflect the correlation between the transcriptomes of human and mouse tissues. Our hypothesis is that, in the same types of tissues, orthologous genes are expressed in a correlated fashion at the global level in both species. The systematic shift of the locations between corresponding human and mouse tissue clusters may be explained by platform effects that remain after data normalization or it may reflect the genuine difference in expression patterns between the species.

Samples such as mammary gland and hematopoietic system were removed from the analysis presented in Figure 2 and Additional file 4 due to their one-sided presence in one species. Our initial PCA studies included these samples; the overall landscape of the PCA plot was different from what we have seen so far but the clustering of samples from nervous system, samples from muscle and heart, as well as the resemblance of such clusters between human and mouse is still evident (Additional file 5). Thus, we believe that the cross-species global gene expression similarity we observed is not due to sample filtering.

It is interesting to observe that all mouse clusters are closer to the center than their human counterparts (Figure 2; Additional files 4 and 5). The observation may reflect that the expression values on the mouse chip are not as widely diversified as those on the human chip; or may simply reflect that the mouse dataset scaled differently to the human dataset during normalization.

How the data were normalized before they were merged into a combined matrix has profound impact on the PCA landscape. In all PCA results we presented so far, the data were normalized by probeset across all samples to minimize the platform differences among samples; thus, the data are more comparable cross-species. If we normalized the human and mouse data matrices by sample, in the combined matrix, the platform difference is the largest variance captured in the top principal component (Additional file 3b), separating mouse samples and human samples into two distinctive areas (Additional file 6a). Within each species cluster, the tissue clusters are still preserved and the relative order of the tissue clusters is the same in the two species (Additional file 6b), reflecting the global gene expression resemblance of the two species.

**Figure 3:**
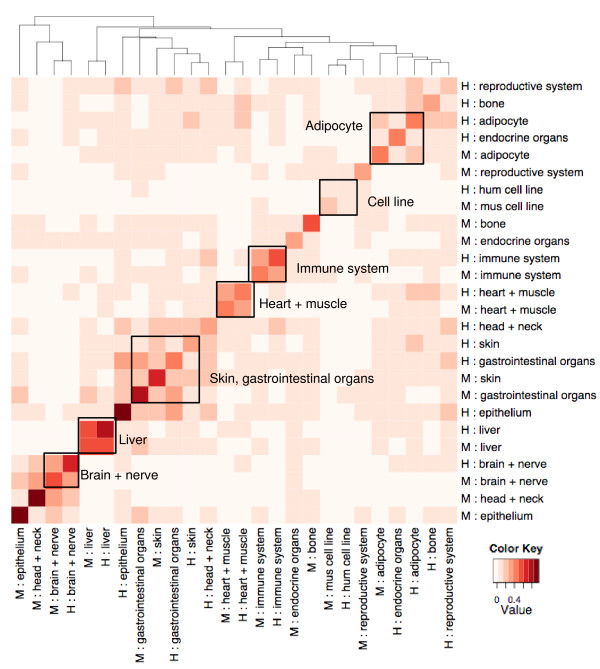
**Hierarchical clustering heatmap of Pearson correlation coefficients between major tissue types of human and mouse**. The outlined boxes indicate tissues in which human and mouse data clustered together.

The similarity between the human and mouse tissue clusters observed on PCA plots is also observed after hierarchical clustering of sample groups. A Pearson correlation coefficient matrix between 26 categories of tissues (13 for human and the same 13 for mouse) was hierarchically clustered (see Materials and methods for details). For liver, muscle/heart, nervous system, cell lines, adipocyte tissues, immune system, skin and gastrointestinal organs, human and mouse data clustered side by side on both X and Y axis (Figure 3). Within such tissue clusters of human and mouse, while the same tissue of the same species displays the highest correlation of gene expression levels, the same tissue of different species often has a higher correlation of gene expression levels than background away from the diagonal. Such cross-specifies correlation is seen in a similar heatmap with a more detailed tissue annotation (Additional file 7).

### Identification of expression correlation between orthologous genes of different species

Cross-platform comparison of gene expression data is always a challenge. Even for the same tissue type, human and mouse samples differ in many ways; thus, it is difficult to take a pair of orthologous genes between the two species and compare their expression levels directly. A condition that induces or suppresses the expression of a gene in one species may not be applicable to another species. To minimize sample and platform variations, we used a measurement called correlation of correlation coefficient or corCor [28]. It compares transcriptome-wide correlation in two groups of corresponding probesets by calculating the vector of correlation coefficients for one probeset to all other probesets in each of the two groups separately, then calculating the correlation coefficient between these two vectors. In our study, the mouse data matrix of 1,267 samples and 6,180 probesets and the human data matrix of 2,557 samples and 6,180 probesets were compared by calculating corCor for every probeset (see Materials and methods). As a negative control, the expression values in the mouse and human data matrices were randomized and the corCor for each probeset was calculated between mouse and human.

**Figure 4:**
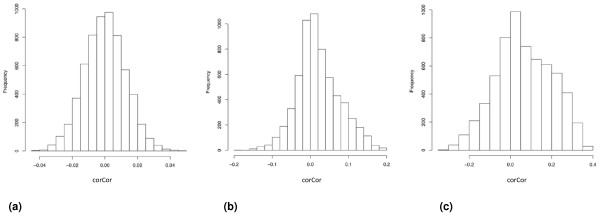
**Distribution of corCor between human and mouse ortholog genes. X-axis is corCor value; Y-axis is number of orthologs**. **(a) **Randomized negative control. **(b) **corCor between human genes and their mouse orthologs in all samples. **(c) **Positive control with corCor between human genes measured in nervous system and human genes measured in muscle/heart. Please note that the values on the X-axis in (b,c) are a magnitude higher than those in (a).

The distribution of corCor for all 6,180 probesets shows that orthologous genes have high corCor compared to a negative control (Figure 4a,b): in the test group, 599 genes had corCor >0.1; in the negative control no gene had corCor >0.05, suggesting, when we look at the data globally taking all tissue types in consideration, a fraction of human and mouse orthologs are expressed in a correlated way. The corCor quantity was also calculated in a positive control comparing 233 human muscle and heart samples with 411 human nervous system samples (Figure 4c). As can be assumed, human genes in different human samples exhibit higher between-group correlations than human genes and mouse orthologous genes.

In contrast to what we observed in Figure 4b, when corCor was measured between mouse and human samples within specific tissues, corCor distributions are not strongly deviating from the negative control (Additional file 8). We believe when samples are of a single tissue type and relatively homogenous, the platform effects and laboratory effects become more dominant and can mask the tissue-specific global expression patterns observed in analyses using much larger and heterogeneous datasets.

**Figure 5:**
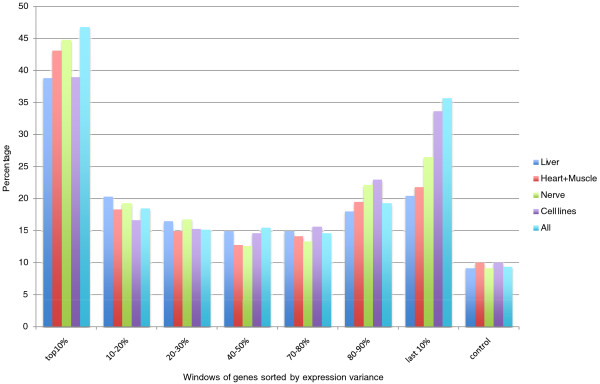
**Percentage of shared mouse and human orthologs in windows of 600 genes sorted by expression variance (descending from left to right)**.

Since corCor is not suitable to identify correlating human and mouse genes at the tissue level, an alternative approach was attempted to identify orthologous genes that are expressed in a correlated fashion in the two species. The expression variance of every gene was calculated one tissue and one species at a time. For each tissue type, the genes are sorted based on their variance. When comparing the sorted gene lists for a human tissue and its corresponding mouse tissue, we observed that, on average, 42% of the most variable 600 genes in one species have ortholog counterparts in the most variable 600 genes in the other species (Figure 5; Additional file 9). For the 600 least variable genes, this figure is 27%. This enrichment of orthologs in highly and lowly variable genes is present in all four tissue types that have segregating clusters in the PCA analysis - liver, nervous system, muscle/heart, and cell lines, as well as in the set of all samples combined and analyzed together. As a negative control, the data were randomized by shuffling the expression values in the data matrices and the percentage of overlapping ortholog pairs is, on average, 10% for all tissues and all variance windows we tested. It is clear that a human tissue and its corresponding mouse tissue share through orthology a good fraction of the most variable genes (tissue-specific genes) and the most constant genes (housekeeping genes); the level of sharing is as strong as the level of human genes co-vary between two different human tissues, which is also around 40% for the top 10% most variable genes (Additional file 9). Data used for this analysis can be found on our web site [27].

A simple binary test done by Chan *et al. *[6] also identified close to 400 1-1-1-1-1 orthologous genes across vertebrate clades that display conserved expression in at least one of ten tissues they tested at the most stringent threshold. To see how many genes the two studies identified as those with evolutionarily conserved expression profile overlap, we created two lists: a list of 273 orthologs we identified as expressed in the nervous system of both human and mouse with top10% variance, and a list of 110 genes that are expressed in the nervous system of all 5 species tested by Chan *et al. *at the highest threshold (top 1/6). We identified 13 overlap genes between the two lists. Our study used 6,108 orthologs, whereas Chan's study used 3,074, with an overlap of 1,344 genes. Of the 273 genes we identified, 51 are in the 1,344-gene set, and of the 110 genes Chan *et al. *identified, 79 are in the same 1,344-gene set. A simple hypergeometric probability test shows that the chance of having 13 overlaps between 51 and 79 genes randomly taken from a common pool of 1,344 genes is low (*P *= 2.9 × 10^-6^), suggesting the overlap of the results from the two studies is significant. The same comparison was also done in heart/muscle and liver; similar overlaps with more significant *P*-values were observed between the two methods, showing significant overlap between gene sets identified by the two studies (Table 2).

**Table 2:** Comparison of the lists of genes that display the evolutionarily conserved expression patterns in different tissues as identified by us and by Chan and colleagues [6]

Tissue	Study	Conserved probesets	Conserved genes	Conserved genes in the common list	Overlaps	*P*-value
Heart/muscle	This study	259	260	49	17	1.8 × 10^-8^
	Chan *et al. *[6]	NA	141	101		
Liver	This study	233	244	40	13	2.3 × 10^-7^
	Chan *et al. *[6]	NA	106	83		
Nervous system	This study	269	273	51	13	2.9 × 10^-6^
	Chan *et al. *[6]	NA	110	79		

The functions of the enriched human mouse orthologs were examined by studying Gene Ontology (GO) term over-representation in the gene list using ONTO-EXPRESS [29]. ONTO-EXPRESS uses the ontology tree and calculates statistical significance for each biological process as *P*-values. We found that the most variable genes shared by human and mouse tend to be genes with tissue-specific functions. For instance, for nervous system samples, the shared gene list contains genes involved in nervous system development and synaptic transmission (Additional file 10a). For muscle and heart samples, the over-represented GO terms in the most variable genes are muscle development, regulation of striated muscle contraction, ventricular cardiac muscle morphogenesis, cardiac muscle contraction, muscle filament sliding, and actin filament-based movement (Additional file 10b). For liver samples, liver-specific GO terms such as oxidation-reduction, lipid metabolic process, response to mercury ion, and cholesterol homeostatasis are enriched (Additional file 10c). This leads to the conclusion that genes with evolutionarily conserved expression patterns across species are mostly the ones performing highly tissue-specific functions and are expressed in specific tissues with limited cell types. This explains the observation made by others [6] and us that tissues with relatively homogenous composition of cell types, such as heart/muscle, liver, and nervous system, would be segregated when profiling large-scale gene expression data. On the other hand, the shared orthologs among the least variable genes tend to be housekeeping genes, such as genes controlling transcription, apoptosis, cell adhesion, cell differentiation and protein amino acid phosphorylation (Additional file 10d). Not surprisingly, the housekeeping genes are also expressed in a similar manner across species.

## Conclusions

With large amounts of gene expression data obtained from public repositories, we investigated the transcriptomes of human and mouse across a large variety of experimental conditions. Where single experiments benefit from reducing experimental variability to discover gene-specific expression regulation, by instead selecting as wide a variety of experimental and sample conditions as possible, we can gain insights into regulation at a higher level of complexity. When analyzing samples from a large variety of tissues, such large-scale studies revealed that the patterns of global gene expression are strong enough to segregate samples based on key biological properties, despite vast variations in experiment conditions, genetic background, age, sex and other sample characteristics. The results confirmed the common belief that samples of similar tissue types share similarities at the transcriptome level. At the same time, the patterns of this segregation, as detected by PCA, are similar between mouse and human and indicate that, on a global level, the signals driving tissue specificity are similar between the species. It supports previous findings [6-9] that although mechanisms of individual gene regulation may be different between the species, global functional patterns are similar and identifiable with whole transcriptome analysis. In particular, like in our study, Chan and colleagues [6] observed in a cross-species comparison of five different vertebrates ranging from human to pufferfish that the expression profiles of orthologous genes across the five species in related tissues of different species were conserved; among other tissues, they also identified heart/muscle, central nervous system and liver as tissues with evolutionarily conserved gene expression profiles [6].

Our results provide strong evidence that, on a global level, gene expression patterns of human-mouse orthologs are conserved. The cross-species conservation of expression profiles of tissue-specific genes and housekeeping genes is the foundation for the similar landscapes of sample clustering between human and mouse in large-scale transcriptome comparison. A recent publication [30] documents that approximately half of measured subnetworks of transcription factors are conserved between human and mouse; this may at least partially explain the conservation of global gene expression patterns we observed in this study.

## Materials and methods

### Creating an integrated mouse gene expression dataset

We identified 2,290 CEL files generated on Affymetrix chip MG_U74Av2 from ArrayExpress; these are all from publicly available experiments deposited to ArrayExpress before May 2008. The quality of the CEL files was evaluated individually using the R *simpleaffy *package and four quality control measurements were produced: average background (AvgBg), scale factors (sfs), percent present (PP) and RNA degradation slope (RNAdeg). Arrays were selected for inclusion in this study based on these quantities using the following ranges: AvgBg, 20 to 150; PP, 25 to 65; RNAdeg, <1.7; sfs, 0.1 to 2.5 (suggested by [31]).

In addition to the *simpleaffy *assessments, the CEL files selected were further evaluated by probe level model (PLM) using the Bioconductor's *affyPLM *package. Two quality assessments were derived from the PLM fitting output: normalized unscaled standard error (nuse) and relative log expression (rle). The cutoffs were set as: nuse, 0.97 to 1.05; rle, -0.15 to 0.15. Arrays not passing these criteria were discarded from further analysis.

The resulting 1,323 CEL files were pre-processed using Bioconductor's *RMA *package [32] to create an integrated, normalized data matrix. Annotations for each sample were retrieved from the database and manually curated to ensure uniform representation and minimal redundancy. For instance, when in some experiments samples were originally annotated as 'hepatocyte samples', we would change the annotation to 'liver' for consistency. The annotations of the 1,323 samples were generalized so the whole dataset contains a limited number of unique categories of tissue type annotation, such as nervous system, reproductive system, immune system and so on. The integrated dataset was submitted to ArrayExpress and assigned accession [E-MTAB-27].

### Merging human and mouse gene expression datasets

The high quality CEL files of 5,372 human samples tested on the HG-U133A microarray were selected and prepared as previously described [25]. The high quality CEL files for mouse samples were selected as described above. The data were normalized separately for human and mouse in R using the *justRMA *function. In the resulting matrices, each column contains data for one sample and each row data for one probeset. The two matrices were then reduced to a subset of probesets representing orthologous genes between mouse and human. The pairing of these orthologous probesets was done based on gene orthologs obtained from Ensembl Compara [33]. Since the probe effect is well known to be very significant in all microarray analyses, we chose to identify orthologous probesets by maximizing the number of probes with similar sequences as follows. For each orthologous gene pair, data for all probesets and their associated probes and probe sequences were retrieved from Affymetrix. Probes for each human gene were BLASTed against mouse probes of the corresponding orthologous gene using bl2seq, and the best one-to-one match was retained. Default settings were used with bl2seq except -W 7, -G 5, -E 2, -F = F. The human-mouse probeset pair with the most probe-probe top matches was selected to represent the ortholog pair on the probeset level.

After we discarded rows with non-orthologous probesets from the human and mouse matrices, the remaining data on each matrix were normalized either by probeset or by sample. To normalize by probeset, we first centered data row by row on median zero by subtracting the row median from each value in the row. Then the centered values were divided by median absolute deviation to scale the data. To normalize by sample, we used the same procedure but centered and scaled the data by columns instead of by rows; column median was used to center the data and column median absolute deviation was used to scale the data. After normalization either by probeset or by sample, the two data matrices of centered and scaled values were merged into one matrix by concatenating the sample columns of orthologous probesets. In the merged matrix, the rows are probesets and the columns are human and mouse samples.

### Principal component analysis

PCA is a technique that transforms a dataset onto a linear space spanned by a number of orthogonal components, ordered by decreasing variance of the data when projected on it. The technique facilitates dimensionality reduction and noise filtering by the projection of data onto a number of the principal components, maximizing the variance retained. The function *prcomp *with default settings provided in the R statistic package was used to perform PCA on different data matrices throughout this study. The results were visualized by scatter plots.

### Hierarchical clustering

The combined data matrix of 2,557 human samples and 1,267 mouse samples created as described above was used for hierarchical clustering. The matrix contains gene expression values centered and scaled by probeset. Each sample in the matrix is assigned to one of 13 general tissue categories that are well represented in both species so the total number of annotation types is 26 (tissue combining species). We extracted 26 submatrices containing data from samples of 26 different annotation types; Pearson correlation coefficients were calculated for 26 × 26 permutations of the submatrices; for each pair of submatrices, a mean correlation coefficient was taken and placed in a 26 × 26 matrix. Hierarchical clustering of the samples in the matrix was performed by R function *heatmap.2*.

### Calculation of corCor

For a gene A on the human array composed of n genes, we computed its pair wise Spearman correlation coefficient with every gene on the same chip, giving a vector v(A) of length n - 1. Given gene A' is the ortholog of gene A on the mouse array, we similarly computed its pair wise correlation coefficient with every mouse gene as v(A') of length n - 1. The correlation coefficient between v(A) and v(A'), corCor, provides an indication of whether A and A' are correlated in mouse and human on the transcriptome level, regardless of the vast sample variations. The higher the absolute corCor value, the stronger correlation of the orthologous genes is; negative corCor indicates negative correlation. The R package *MergeMaid *was used for this analysis [34].

## Abbreviations

corCor: correlation of correlation coefficient; GO: Gene Ontology; PCA: principal component analysis; PLM: probe level model.

## Competing interests

The authors declare that they have no competing interests.

## Authors' contributions

XZ designed and carried out all analyses and wrote the manuscript. JR participated in the design and interpretation of the study and contributed to manuscript writing. HP participated in the design and coordination of the study. AB conceived the study and participated in its design and helped to draft the manuscript. All authors read and approved the final manuscript.

## Authors' information

AB is a senior team leader and senior scientist at EMBL-EBI and serves on the board of FGED (Functional Genomics Data) Society.

## Supplementary Material

Additional file 1 **PCA plot of the integrated mouse gene expression data matrix**. The two axes are components 2 and 3; each dot represents a sample, colored by experiment accession number. While experiments with more than 15 samples are labeled as individual experiments, experiments with smaller numbers of samples are grouped into one category, 'small exp' (light brown). Tissue clusters observed in Figure 1 are circled. No apparent clustering of samples based on experiments is observed. Click here for file

Additional file 2 **Experiments and samples used for the mouse PCA**. Click here for file

Additional file 3 **Distribution of gene expression variances for the top 50 principal components**. The histograms were plotted for PCA results of the combined human mouse data matrix normalized by **(a) **probeset or **(b) **sample. Click here for file

Additional file 4 **PCA plot of a combined human and mouse gene expression data matrix (principal components 1 and 2)**. The samples are labeled by **(a) **species and **(b) **tissue type. Four major sample clusters are indicated: muscle/heart samples (red), nervous system samples (blue), liver samples (purple) and cell line samples (green). For these clusters, human and mouse samples exhibit subclustering in proximity to each other. Click here for file

Additional file 5 **PCA plots of a combined human and mouse gene expression data matrix with all samples**. The samples are labeled by **(a) **species and **(b) **tissue type. Unlike previous PCA plots, samples such as mammary gland and hematopoietic system whose presentation is mostly one-sided in one species were removed from the analysis; this PCA included all high quality data from both human and mouse. The clustering of samples from nervous system (green), muscle/heart (lilac), cell lines (brown), and liver (pink) is still evident among the overwhelmingly dominant hematopoietic samples (blue) and mammary gland samples (turquoise). The corresponding human and mouse sample clusters resemble each other. Samples of unknown tissue type annotation are colored white and labeled as '0'. Click here for file

Additional file 6 **PCA plots of a combined human and mouse gene expression data matrix normalized by sample**. The samples are labeled by **(a) **species and **(b) **tissue type. Mouse samples (black) and human samples (red) are well separated on the axis of component 1. Tissue clusters in the two species are projected to the second principal component in a similar order: nervous system (blue), muscle/heart (red), liver (purple) and cell lines (green). Click here for file

Additional file 7 **Hierarchical clustering heatmap of Pearson correlation coefficients between different types of tissues in human and mouse**. Tissues in which human and mouse data clustered together are outlined by boxes. Click here for file

Additional file 8 **Distribution of corCor between human and mouse ortholog genes in specific tissues**. The X-axis is the corCor value between human and mouse gene expression levels in **(a) **nervous system and **(b) **cell line samples. The Y-axis is the number of orthologs. In these analyses, corCor distribution is not very different from a randomized negative control (Figure 4a). Click here for file

Additional file 9 **Percentage of common genes in the top 10% most variable genes between different tissues of the same species, as well as between different tissues of human and mouse**. The numbers in bold are those represented in the top 10% group in Figure 5. Click here for file

Additional file 10 **Functional analysis of orthologous genes shared between mouse and human in the top 10% most variable genes and the top 10% least variable genes**. **(a-c) **The top 10% most variable genes and **(d) **the top 10% least variable genes: (a,d) nervous system; (b) muscle/heart; (c) liver. In (a-c), GO over-representation was sorted by corrected *P*-value and then by level of GO term enrichment; only the top ten categories are displayed. Genes with tissue-specific functions are colored in orange. The over-represented GO terms in (d) were sorted by count of genes in each category; the top categories are mostly housekeeping molecular functions. Click here for file
