# Supplementary material for: Large scale comparison of global gene expression patterns in human and mouse
Source: Genome Biol. 2010 Dec 23;11(12):R124. doi: 10.1186/gb-2010-11-12-r124 (PMC3046484; doi:10.1186/gb-2010-11-12-r124)
Supplement: Additional file 1 — PCA plot of the integrated mouse gene expression data matrix. The two axes are components 2 and 3; each dot represents a sample, colored by experiment accession number. While experiments with more than 15 samples are labeled as individual experiments, experiments with smaller numbers of samples are grouped into one category, 'small exp' (light brown). Tissue clusters observed in Figure 1 are circled. No apparent clustering of samples based on experiments is observed. [file gb-2010-11-12-r124-S1.ppt]

## Slide 1
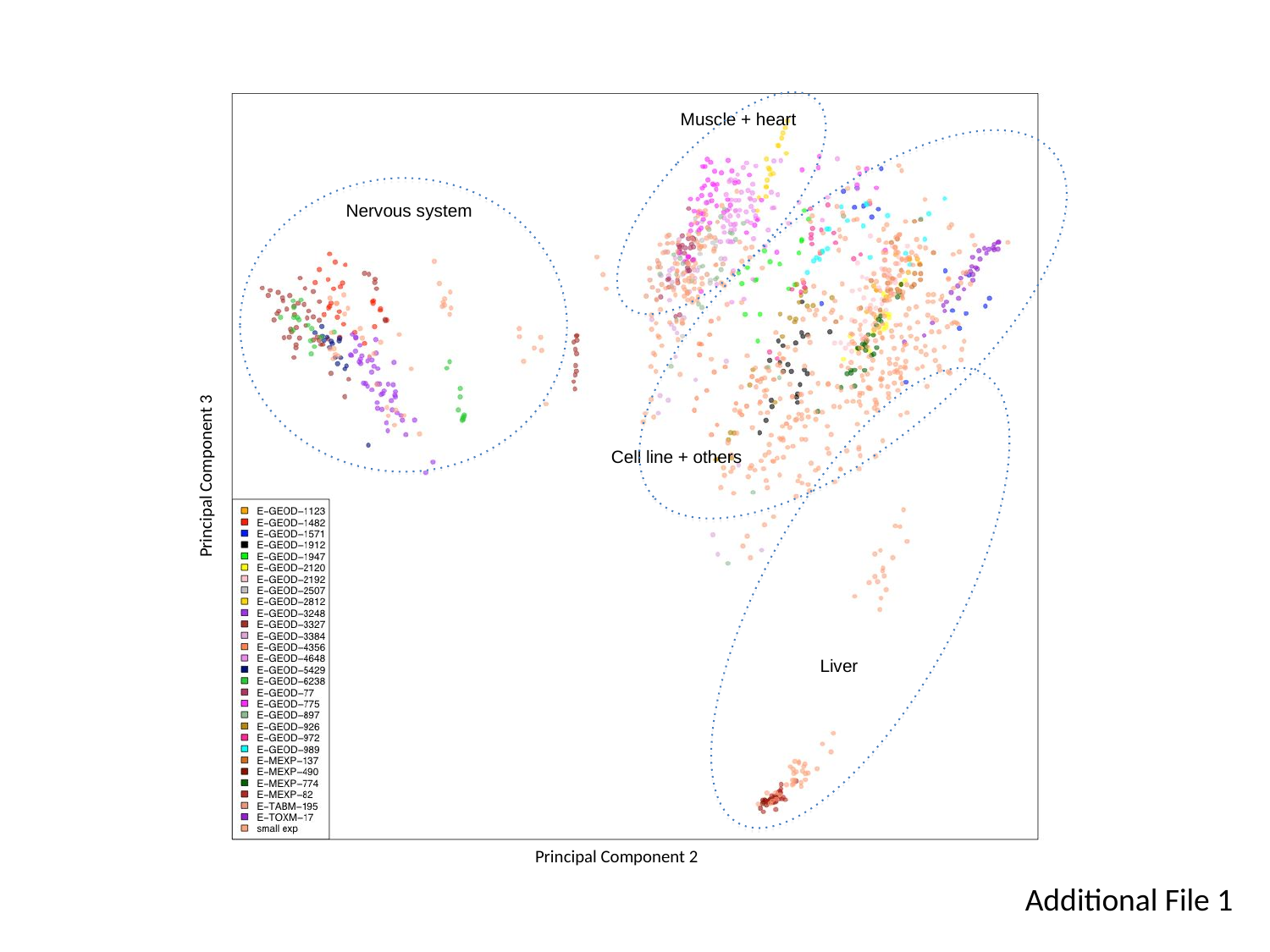

Muscle + heart
Nervous system
Cell line + others
Liver
Principal Component 3
Principal Component 2
Additional File 1
