# Supplementary material for: Large scale comparison of global gene expression patterns in human and mouse
Source: Genome Biol. 2010 Dec 23;11(12):R124. doi: 10.1186/gb-2010-11-12-r124 (PMC3046484; doi:10.1186/gb-2010-11-12-r124)
Supplement: Additional file 2 — Experiments and samples used for the mouse PCA. [file gb-2010-11-12-r124-S2.ppt]

## Slide 1
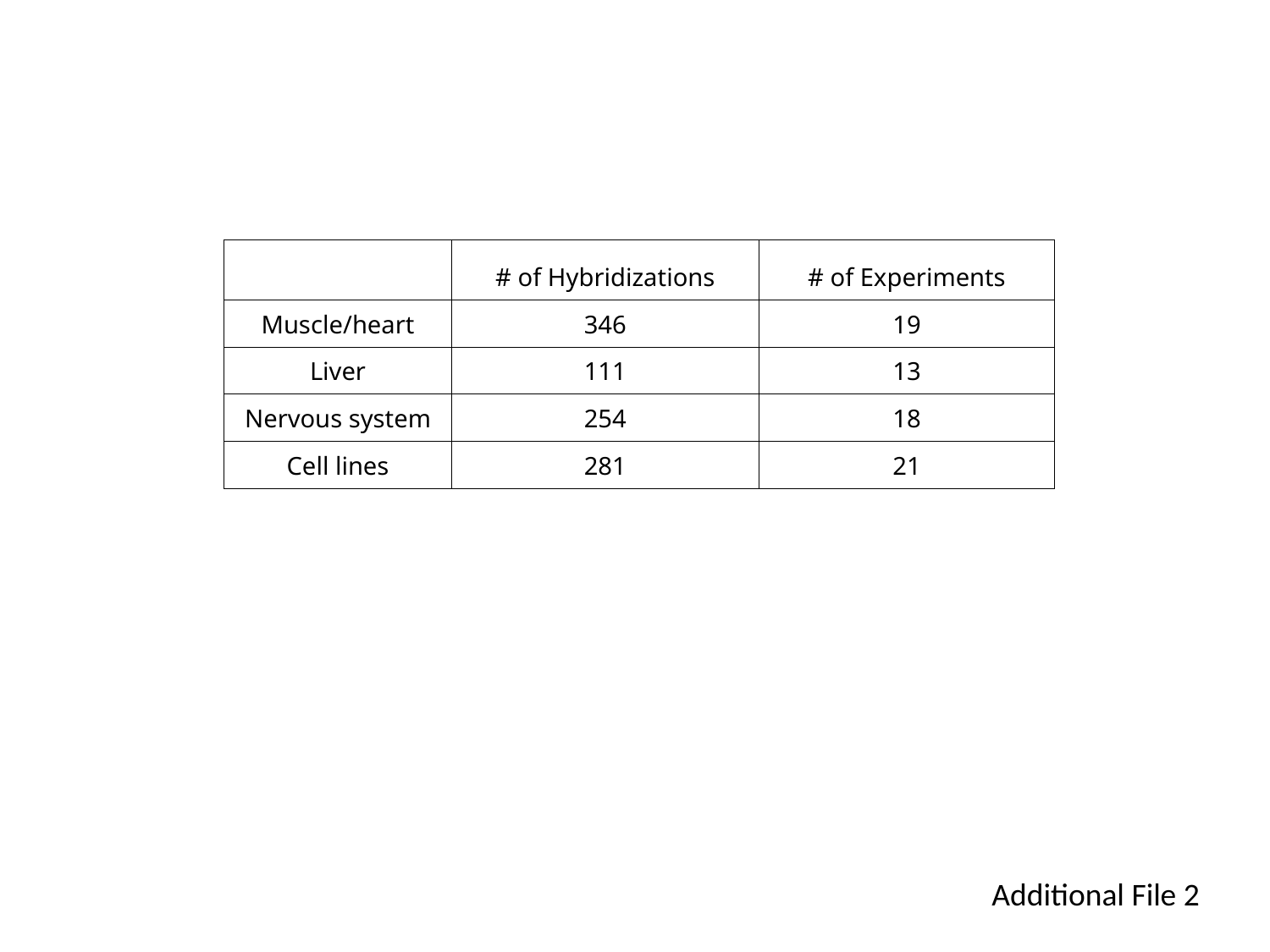

| | # of Hybridizations | # of Experiments |
| --- | --- | --- |
| Muscle/heart | 346 | 19 |
| Liver | 111 | 13 |
| Nervous system | 254 | 18 |
| Cell lines | 281 | 21 |
Additional File 2
