# Supplementary figures and images for: Large scale comparison of global gene expression patterns in human and mouse
Source: Genome Biol. 2010 Dec 23;11(12):R124. doi: 10.1186/gb-2010-11-12-r124 (PMC3046484; doi:10.1186/gb-2010-11-12-r124)

## Slide 1
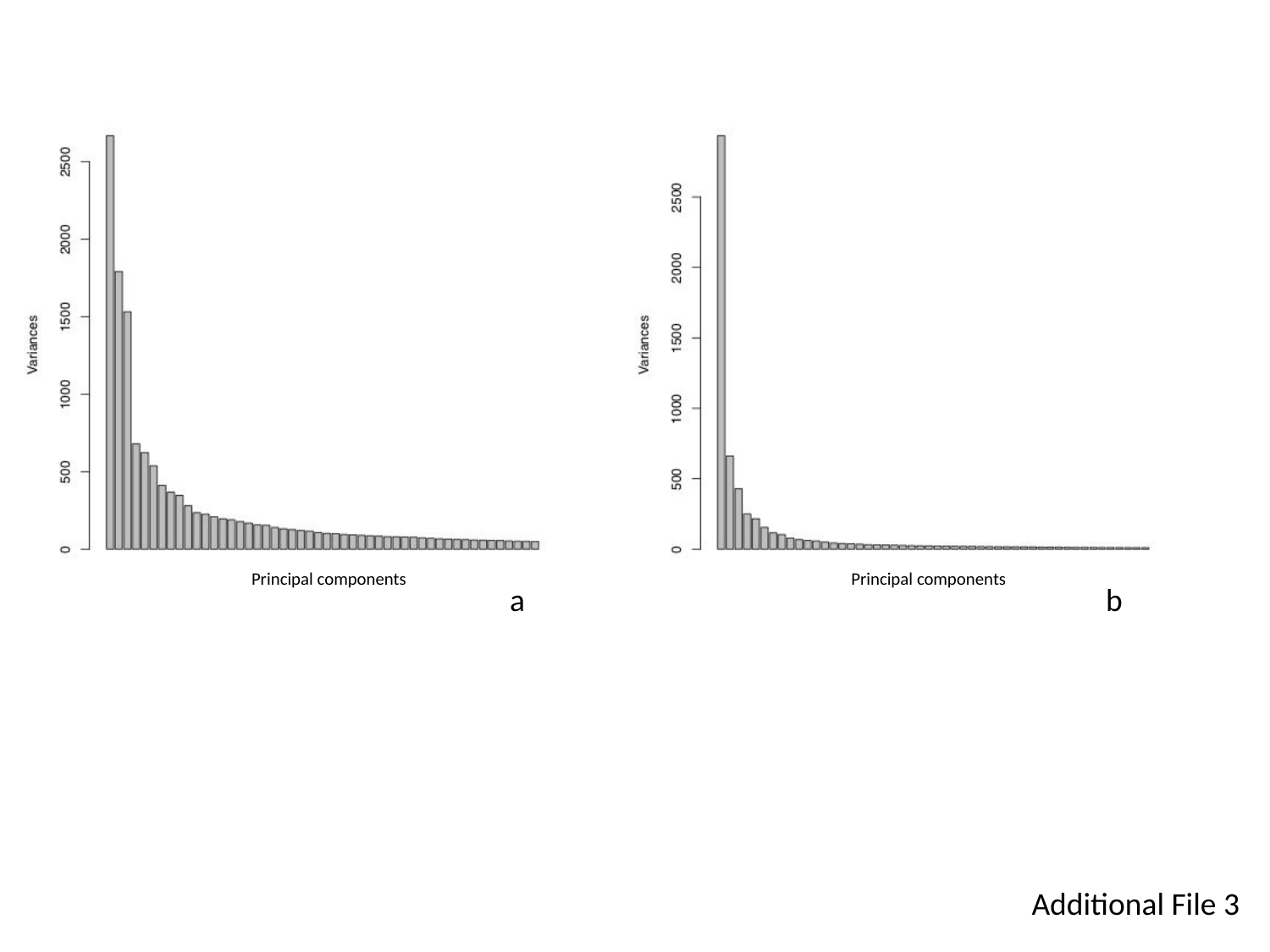

Principal components
Principal components
a
b
Additional File 3

Supplement: Additional file 3 — Distribution of gene expression variances for the top 50 principal components. The histograms were plotted for PCA results of the combined human mouse data matrix normalized by (a) probeset or (b) sample. [file gb-2010-11-12-r124-S3.ppt]

## Slide 1
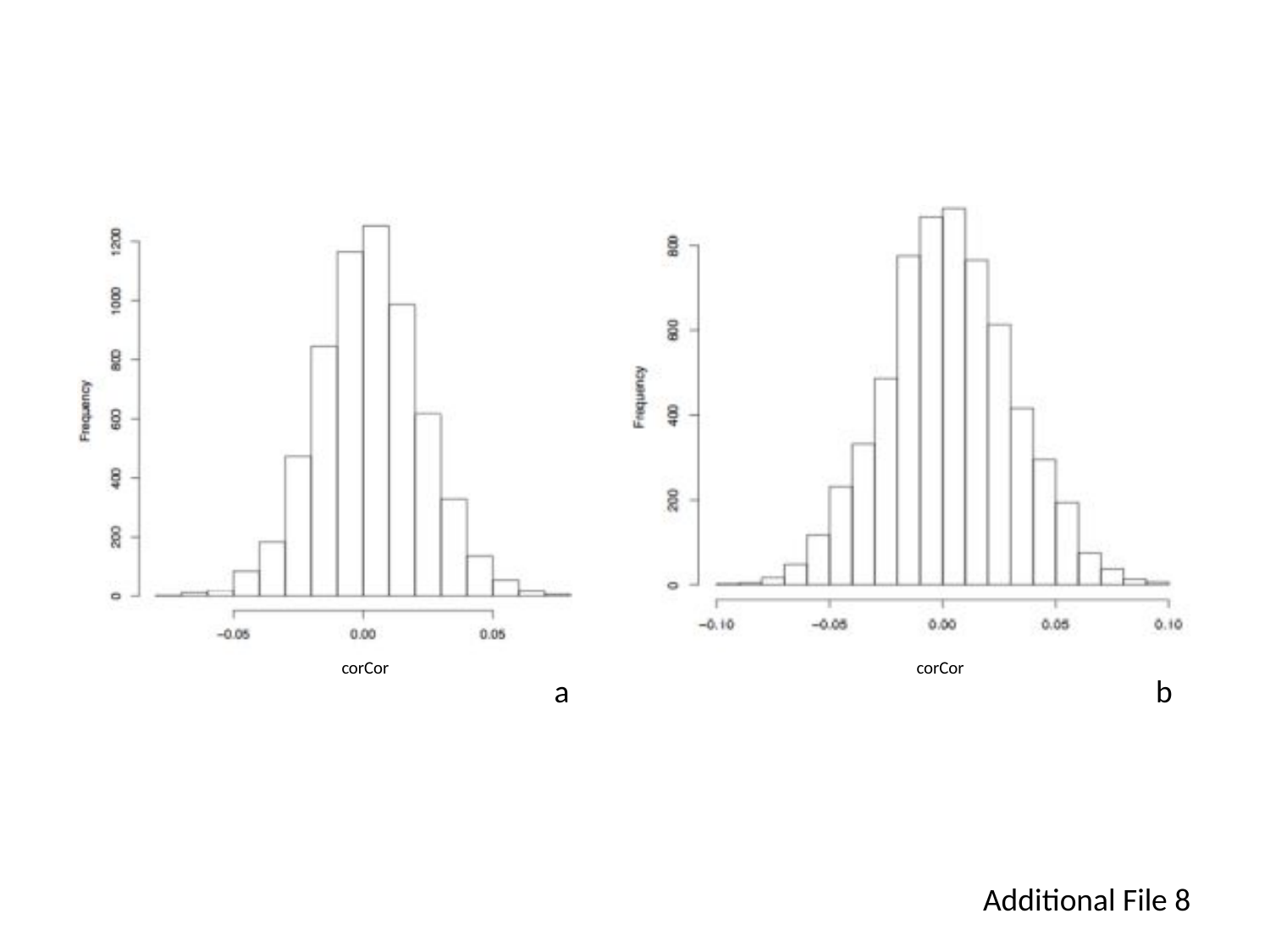

corCor
corCor
a
b
Additional File 8

Supplement: Additional file 8 — Distribution of corCor between human and mouse ortholog genes in specific tissues. The X-axis is the corCor value between human and mouse gene expression levels in (a) nervous system and (b) cell line samples. The Y-axis is the number of orthologs. In these analyses, corCor distribution is not very different from a randomized negative control (Figure 4a). [file gb-2010-11-12-r124-S8.ppt]

## Slide 1
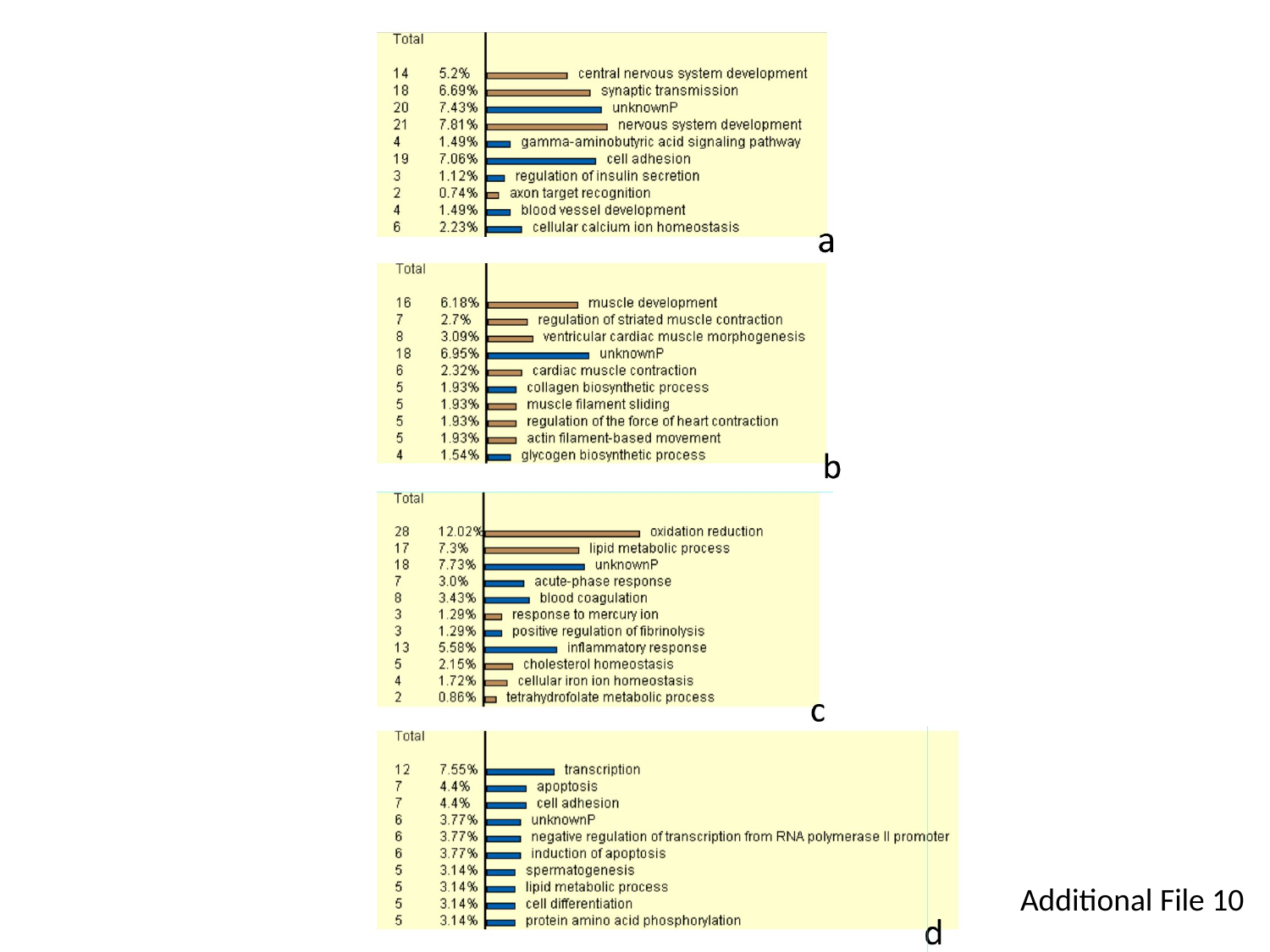

Additional File 10

Supplement: Additional file 10 — Functional analysis of orthologous genes shared between mouse and human in the top 10% most variable genes and the top 10% least variable genes. (a-c) The top 10% most variable genes and (d) the top 10% least variable genes: (a,d) nervous system; (b) muscle/heart; (c) liver. In (a-c), GO over-representation was sorted by corrected P-value and then by level of GO term enrichment; only the top ten categories are displayed. Genes with tissue-specific functions are colored in orange. The over-represented GO terms in (d) were sorted by count of genes in each category; the top categories are mostly housekeeping molecular functions. [file gb-2010-11-12-r124-S10.ppt]
