# Supplementary material for: Large scale comparison of global gene expression patterns in human and mouse
Source: Genome Biol. 2010 Dec 23;11(12):R124. doi: 10.1186/gb-2010-11-12-r124 (PMC3046484; doi:10.1186/gb-2010-11-12-r124)
Supplement: Additional file 4 — PCA plot of a combined human and mouse gene expression data matrix (principal components 1 and 2). The samples are labeled by (a) species and (b) tissue type. Four major sample clusters are indicated: muscle/heart samples (red), nervous system samples (blue), liver samples (purple) and cell line samples (green). For these clusters, human and mouse samples exhibit subclustering in proximity to each other. [file gb-2010-11-12-r124-S4.ppt]

## Slide 1
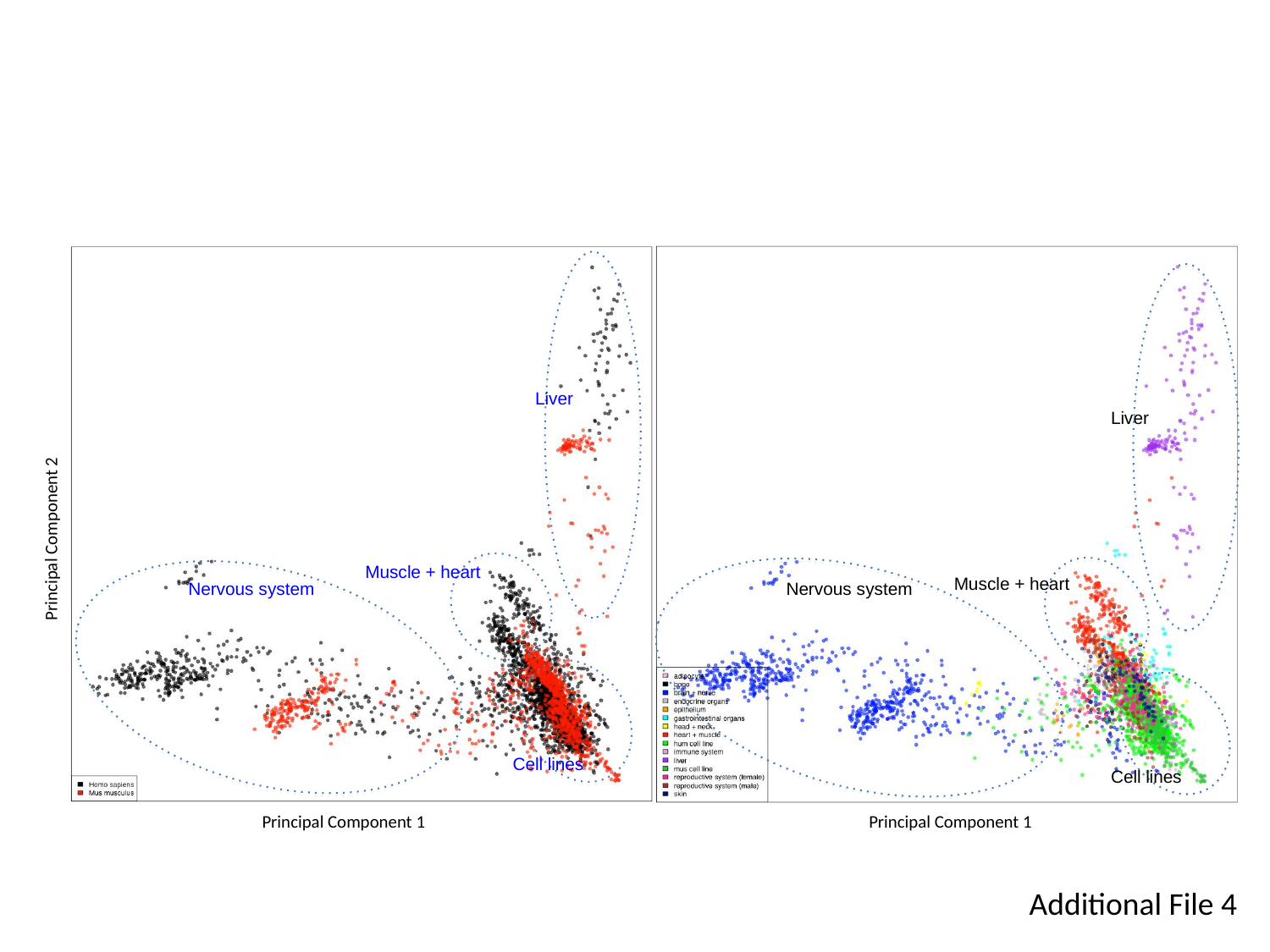

Liver
Liver
Principal Component 2
Muscle + heart
Muscle + heart
Nervous system
Nervous system
Cell lines
Cell lines
Principal Component 1
Principal Component 1
Additional File 4
