# Supplementary material for: Large scale comparison of global gene expression patterns in human and mouse
Source: Genome Biol. 2010 Dec 23;11(12):R124. doi: 10.1186/gb-2010-11-12-r124 (PMC3046484; doi:10.1186/gb-2010-11-12-r124)
Supplement: Additional file 5 — PCA plots of a combined human and mouse gene expression data matrix with all samples. The samples are labeled by (a) species and (b) tissue type. Unlike previous PCA plots, samples such as mammary gland and hematopoietic system whose presentation is mostly one-sided in one species were removed from the analysis; this PCA included all high quality data from both human and mouse. The clustering of samples from nervous system (green), muscle/heart (lilac), cell lines (brown), and liver (pink) is still evident among the overwhelmingly dominant hematopoietic samples (blue) and mammary gland samples (turquoise). The corresponding human and mouse sample clusters resemble each other. Samples of unknown tissue type annotation are colored white and labeled as '0'. [file gb-2010-11-12-r124-S5.ppt]

## Slide 1
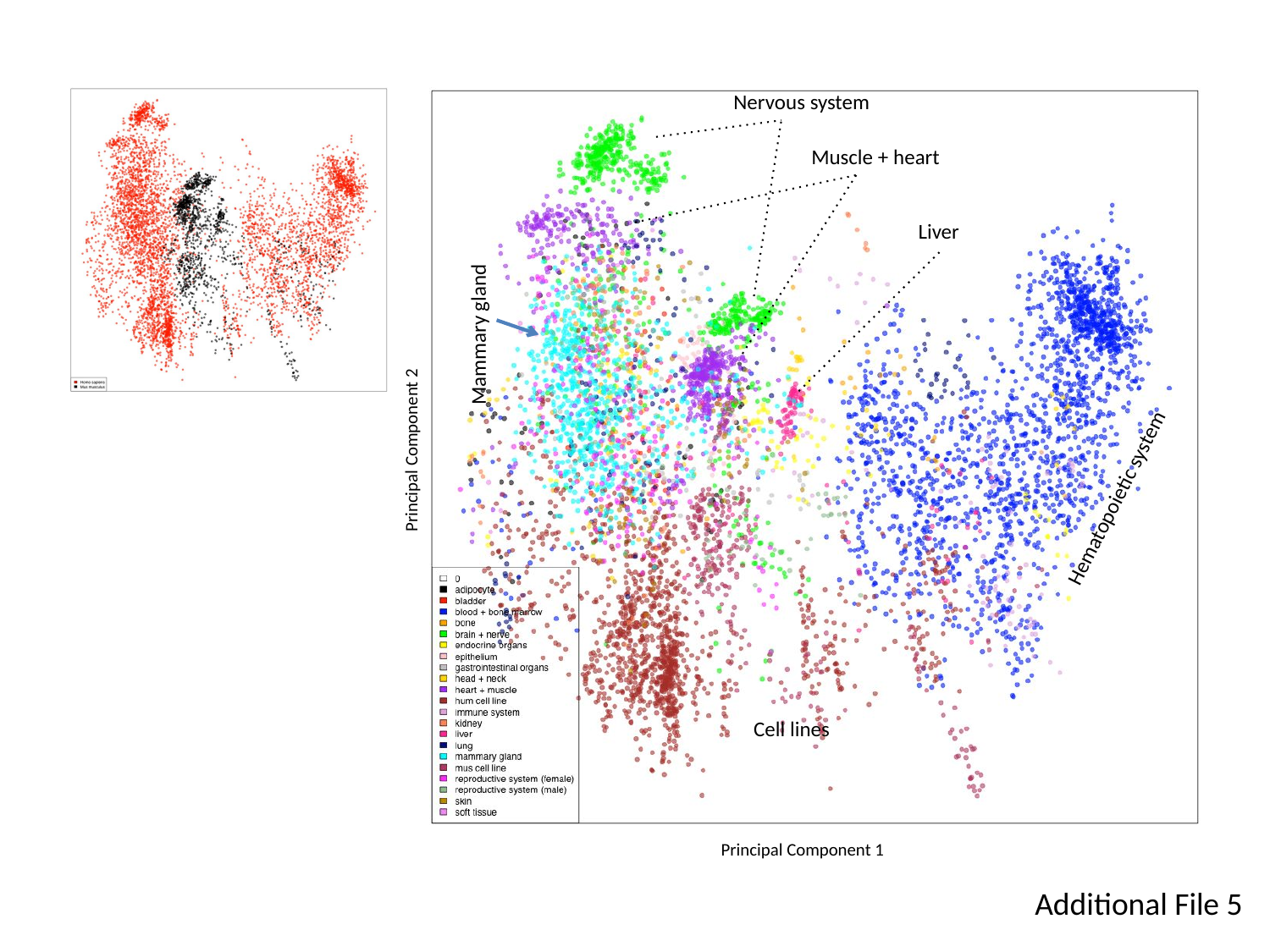

Nervous system
Muscle + heart
Liver
Mammary gland
Principal Component 2
Hematopoietic system
Cell lines
Principal Component 1
Additional File 5
