# Supplementary material for: Large scale comparison of global gene expression patterns in human and mouse
Source: Genome Biol. 2010 Dec 23;11(12):R124. doi: 10.1186/gb-2010-11-12-r124 (PMC3046484; doi:10.1186/gb-2010-11-12-r124)
Supplement: Additional file 6 — PCA plots of a combined human and mouse gene expression data matrix normalized by sample. The samples are labeled by (a) species and (b) tissue type. Mouse samples (black) and human samples (red) are well separated on the axis of component 1. Tissue clusters in the two species are projected to the second principal component in a similar order: nervous system (blue), muscle/heart (red), liver (purple) and cell lines (green). [file gb-2010-11-12-r124-S6.ppt]

## Slide 1
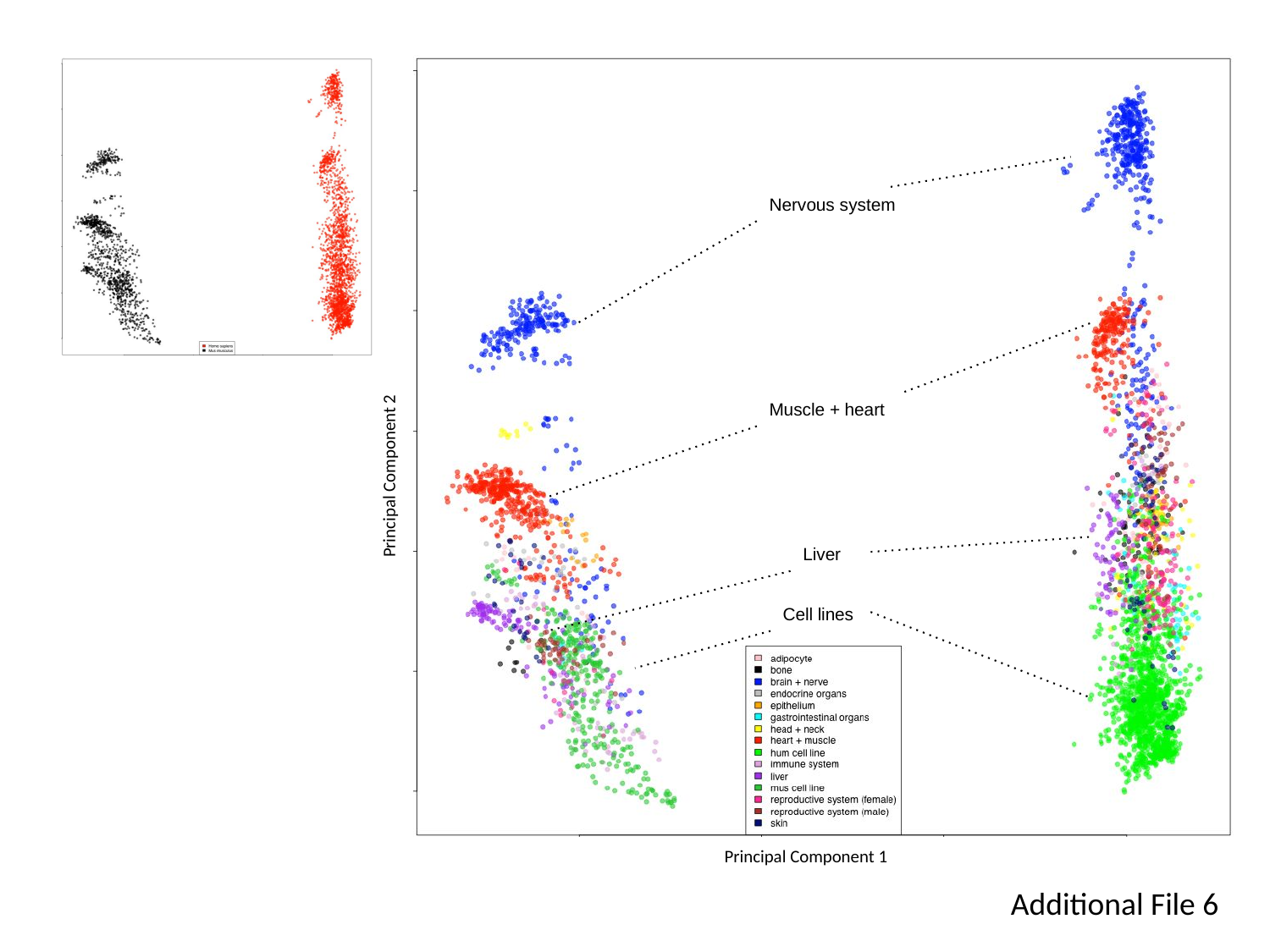

Nervous system
Muscle + heart
Principal Component 2
Liver
Cell lines
Principal Component 1
Additional File 6
