# Supplementary material for: Large scale comparison of global gene expression patterns in human and mouse
Source: Genome Biol. 2010 Dec 23;11(12):R124. doi: 10.1186/gb-2010-11-12-r124 (PMC3046484; doi:10.1186/gb-2010-11-12-r124)
Supplement: Additional file 7 — Hierarchical clustering heatmap of Pearson correlation coefficients between different types of tissues in human and mouse. Tissues in which human and mouse data clustered together are outlined by boxes. [file gb-2010-11-12-r124-S7.ppt]

## Slide 1
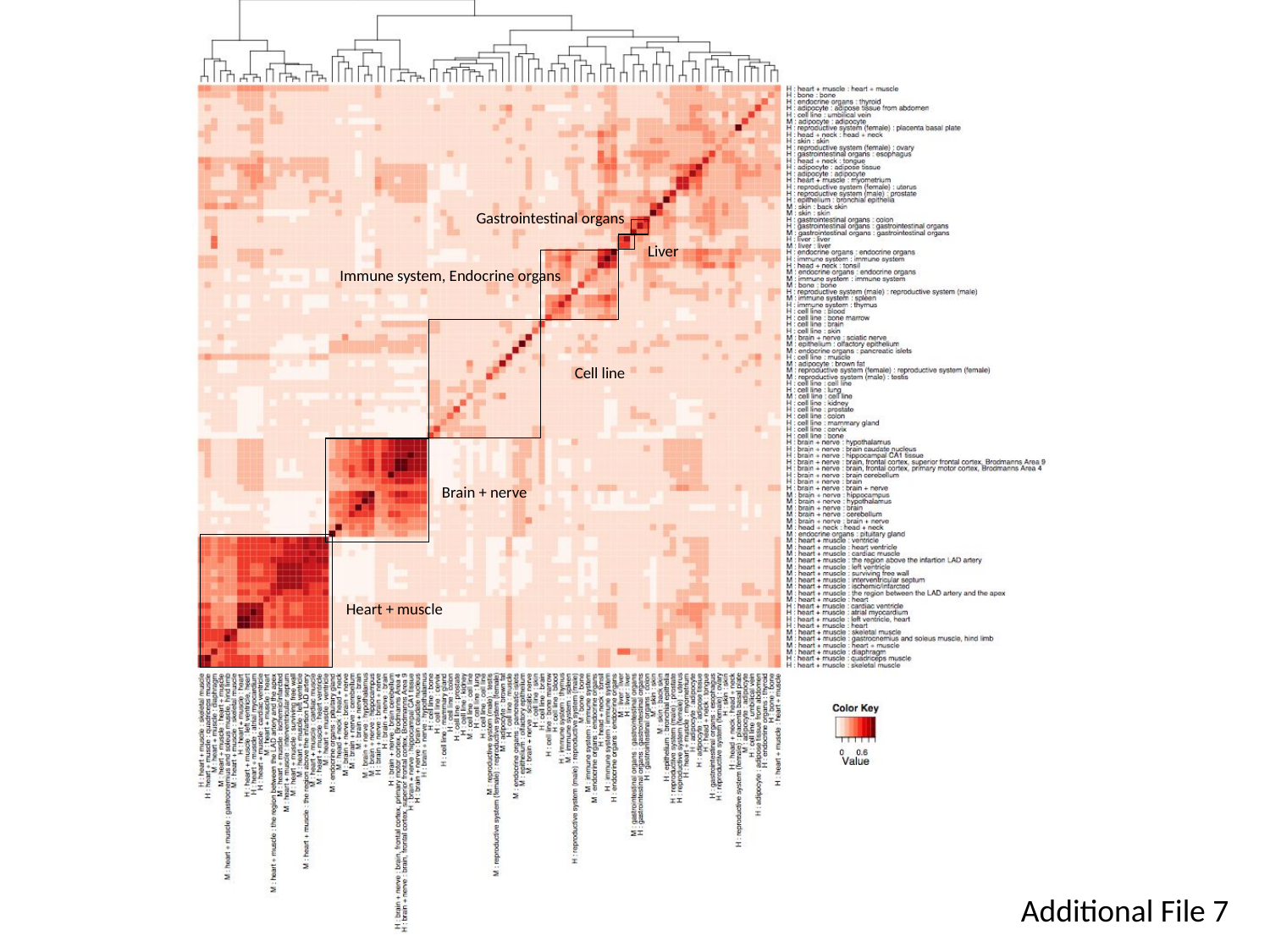

Gastrointestinal organs
Liver
Immune system, Endocrine organs
Cell line
Brain + nerve
Heart + muscle
Additional File 7
