# Supplementary material for: Large scale comparison of global gene expression patterns in human and mouse
Source: Genome Biol. 2010 Dec 23;11(12):R124. doi: 10.1186/gb-2010-11-12-r124 (PMC3046484; doi:10.1186/gb-2010-11-12-r124)
Supplement: Additional file 9 — Percentage of common genes in the top 10% most variable genes between different tissues of the same species, as well as between different tissues of human and mouse. The numbers in bold are those represented in the top 10% group in Figure 5. [file gb-2010-11-12-r124-S9.ppt]

## Slide 1
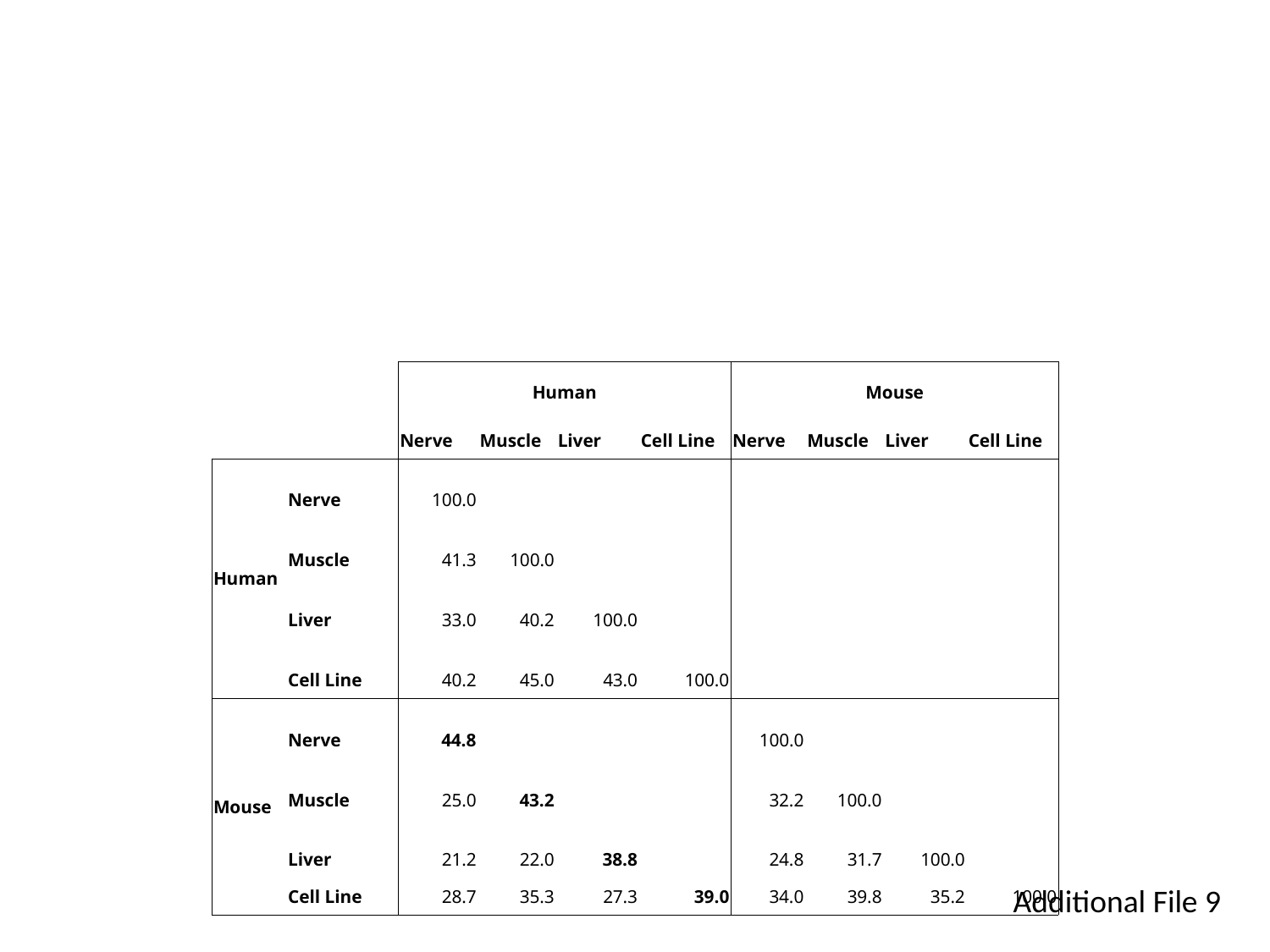

| | | Human | | | | Mouse | | | |
| --- | --- | --- | --- | --- | --- | --- | --- | --- | --- |
| | | Nerve | Muscle | Liver | Cell Line | Nerve | Muscle | Liver | Cell Line |
| Human | Nerve | 100.0 | | | | | | | |
| | Muscle | 41.3 | 100.0 | | | | | | |
| | Liver | 33.0 | 40.2 | 100.0 | | | | | |
| | Cell Line | 40.2 | 45.0 | 43.0 | 100.0 | | | | |
| Mouse | Nerve | 44.8 | | | | 100.0 | | | |
| | Muscle | 25.0 | 43.2 | | | 32.2 | 100.0 | | |
| | Liver | 21.2 | 22.0 | 38.8 | | 24.8 | 31.7 | 100.0 | |
| | Cell Line | 28.7 | 35.3 | 27.3 | 39.0 | 34.0 | 39.8 | 35.2 | 100.0 |
Additional File 9
